# Chemokine Localization in Bronchial Angiogenesis

**DOI:** 10.1371/journal.pone.0066432

**Published:** 2013-06-11

**Authors:** Maria Grazia Perino, Aigul Moldobaeva, John Jenkins, Elizabeth M. Wagner

**Affiliations:** Department of Medicine, Johns Hopkins School of Medicine, Baltimore, Maryland, United States of America; University of Bern, Switzerland

## Abstract

Angiogenesis in the lung involves the systemic bronchial vasculature and becomes prominent when chronic inflammation prevails. Mechanisms for neovascularization following pulmonary ischemia include growth factor transit from ischemic parenchyma to upstream bronchial arteries, inflammatory cell migration/recruitment through the perfusing artery, and paracrine effects of lung cells within the left bronchus, the niche where arteriogenesis takes place. We analyzed left lung bronchoalveolar lavage (BAL) fluid and left bronchus homogenates after left pulmonary artery ligation (LPAL) in rats, immediately after the onset of ischemia (0 h), 6 h and 24 h later. Additionally, we tested the effectiveness of dexamethasone on decreasing inflammation (0–24 h LPAL) and angiogenesis at early (3 d LPAL; bronchial endothelial proliferation) and late (14 d LPAL; blood flow) stages. After LPAL (6 h), BAL protein, total inflammatory cells, macrophages, and polymorphonuclear cells increased significantly. In parallel, pro-angiogenic CXC chemokines increased in BAL and the left main-stem bronchus (CXCL1) or only within the bronchus (CXCL2). Dexamethasone treatment reduced total BAL protein, inflammatory cells (total and polymorphonuclear cells), and CXCL1 but not CXCL2 in BAL. By contrast, no decrease was seen in either chemokine within the bronchial tissue, in proliferating bronchial endothelial cells, or in systemic perfusion of the left lung. Our results confirm the presence of CXC chemokines within BAL fluid as well as within the left mainstem bronchus. Despite significant reduction in lung injury and inflammation with dexamethasone treatment, chemokine expression within the bronchial tissue as well as angiogenesis were not affected. Our results suggest that early changes within the bronchial niche contribute to subsequent neovascularization during pulmonary ischemia.

## Introduction

Angiogenesis in the lung involves predominantly the systemic vasculature and becomes prominent in several pathologic states where chronic inflammation prevails including cystic fibrosis [1], asthma [2], pulmonary fibrosis [3], lung cancer [4] and chronic pulmonary thromboembolic disease [5], [6]. The etiology of this process is largely unknown but proposed mechanisms span the consequences of mechanical stress [7], [8], inflammatory tissue remodeling [9], [10], and growth factor release due to tissue ischemia [11]. Because this vascular expansion of new bronchial arteries is from existing arteries, it is more specifically called arteriogenesis. Very little is known about the potent angiogenic ability of the mature bronchial circulation. Therefore, an overarching challenge is to determine the signaling hierarchy that is stimulated by the need for increased perfusion. Our published work in a rat model has shown that the bronchial circulation undergoes massive proliferation following left pulmonary artery obstruction [12], [13] and that pro-angiogenic cytokines and their G-protein coupled receptors are involved in the overall process. Specifically, the involvement of CXCL2 (CINC-3) and its signaling receptor have been demonstrated, with significant reduction of arteriogenesis following treatment with a CXCR2 neutralizing antibody [12]. Chemokines are expressed by several cell types including neutrophils, macrophages, endothelial and epithelial cells [14]–[16]. However, the precise cadre of molecular initiators of systemic vessel proliferation are unresolved. Moreover, the specific signal localization that drives bronchial arteriogenesis is unknown. Given that parenchymal ischemia takes place with left pulmonary artery obstruction, it is unclear how growth factors drive the orderly spatial and temporal proliferation of the upstream bronchial arteries. Potential mechanisms include growth factor transit from ischemic parenchyma with fluid movement, inflammatory cell migration from ischemic parenchyma or recruitment through the perfusing artery, and a paracrine effect of cells within the left bronchus, the niche where arteriogenesis takes place. The goal of this study was focused on collecting information concerning each of these three potential mechanisms to better understand the early initiators of bronchial arteriogenesis.

Angiogenesis in the lung has been shown to be induced largely during inflammatory states [3], [17], [18]. Unlike other organs where tissue hypoxia plays the predominant role in inducing release of growth factors, the ventilated lung appears to be most sensitive to inflammatory cell burden. Others have shown that treatment with anti-inflammatory agents can limit angiogenesis [19]. The synthetic glucocorticoid dexamethasone has been shown to modulate, among several signaling molecules, pro-inflammatory cytokines such as CCL2 (MCP-1) and sub-members of the CXCL8 family, of which the CINC proteins are members [20], [21]. In the present study we also sought to validate the effectiveness of dexamethasone therapy to negatively modulate the inflammatory response in the ischemic lung and to probe the mechanism of growth factor access to large bronchial vessels. Our results confirm the presence of CXC chemokines within bronchoalveolar lavage fluid as well as within bronchial tissue. Despite significant reduction in lung injury and inflammation with dexamethasone treatment, chemokine expression within the bronchial tissue as well as angiogenesis were not affected. Our results suggest that early changes within bronchial tissue itself contribute to subsequent neovascularization during pulmonary ischemia.

## Methods

### Animals

Our animal protocol was approved by the Johns Hopkins Animal Care and Use Committee (Protocol # RA11M47). Sprague-Dawley male rats (125–150 g; Harlan, Indianapolis, IN) were anesthetized, intubated, and ventilated (90 breaths/min, 8 ml/kg/breath) with an anesthetic-gas mixture (3% isoflurane in O_2_). Left pulmonary artery ligation (LPAL) was performed as previously described [12]. After left lateral thoracotomy between the fourth and the fifth intercostal space, the left pulmonary artery was ligated. The thoracotomy was closed and bupivicaine (2 mg/kg) was injected at the incision site for analgesia. After closing the skin incision with methyl acrylamide adhesive, buprenorphine (0.05 mg/kg ip) was given for additional analgesia, the rat was removed from the ventilator, extubated, and allowed to recover. After specified times following LPAL, anesthetized rats were euthanized by exsanguination.

### Bronchoalveolar lavage (BAL)

Immediately after death, the right lung was isolated and the left lung was washed with room temperature PBS (3×1.0 ml). BAL fluid was gently aspirated, total volume recorded and total cell number counted (Bright Line Hemacytometer; Horsham, PA). Cell differentials were determined by the evaluation of 300 cells/rat (Cytospin 4; Shandon, Pittsburgh, PA and Diff-quick staining; Dade Bering, Newark, DE). Total protein in BAL was measured using a bicinchoninic acid assay (BCA, Thermo Fisher Scientific Inc, Rockford, IL).

### Quantitative real time RT-PCR

Changes in mRNA expression of the chemokines CXCL1 and CXCL2, and their receptors CXCR1 and CXCR2 were evaluated within the bronchial tissue after dissection from lung parenchyma. Left bronchi were mechanically dissociated in TRIZOL (Invitrogen/Life Technologies, Grand Island, NY) and total RNA (0.5 µg) was reverse-transcribed according to manufacturer's protocol (Qiagen, Valencia, CA). Quantitative PCR reactions were performed using QuantiTect SYBR Green PCR Master Mix (Qiagen, Valencia, CA) and CFX96 cycler (Bio-Rad Laboratories, CA), using 1 µl of cDNA as the template in 25 µl reaction mixture. The melting curve protocol was performed following the qPCR to confirm the presence of a single clean melting peak representative of the presence of one single amplicon. Data were normalized to Gapdh mRNA in individual samples.

### Protein

Changes in CXCL1 and CXCL2 at selected time points following LPAL were quantified in BAL and left bronchi by ELISA (RCN100, RCN300, R and D Systems, Minneapolis, MN), according to the manufacturer's protocol. Total protein content was measured by BCA assay. For immunostaining in frozen sections (OCT) of left bronchus, Epcam for epithelium, CXCL2, CXCR2, and RECA-1 for endothelium, were blocked with blocking solution containing goat serum, avidin and biotin (Invitrogen, Grand island, NY) and stained with the antibodies (Epcam/G8.8 clone, Biolegend, San Diego,CA; Gro-beta #ab97777, Abcam,, Cambridge, MA; CXCR2 #ab14935, Abcam, and RECA-1 # MCA970GA, Serotec, Raleigh, NC. After washing, sections were incubated with biotin anti-rabbit antibody followed by streptavidin labeled with Alexa Fluor 488, Alexa Fluor 594 and counterstained with DAPI (all from Invitrogen). Tissue stained without primary antibody was used as control. Sections were visualized and photographed using an Olympus IX51 microscope and SensiCam high performance digital camera (Cooke, Auburn Hills, MI).

### Bronchial endothelial cell proliferation

Morphometric assessment of bronchial endothelial cell proliferation was determined as an index of early angiogenesis 3 d after LPAL. Lungs were fixed by intratracheal infusion of formalin (10%) at 20 cmH_2_O. Serial sections were obtained from 12 different regions of the separated left lung. Bronchial vessels associated with airways were identified in hematoxylin and eosin (H&E) stained sections and companion serial sections were evaluated for Proliferating Cell Nuclear Antigen (PCNA^+^) vessels with the observer blinded to the animal treatment. Blood vessels were scored as showing PCNA positive/negative endothelium. Percent positive vessels were averaged for each lung and considered representative of a specific rat lung.

### Late functional angiogenesis

Systemic blood flow to the left lung was measured 14 d after LPAL using fluorescent microspheres (15 µm; Invitrogen, Eugene, OR). Rats were anesthetized and ventilated as described above, the left carotid artery was cannulated and 500,000 microspheres were infused. Rats were euthanized by exsanguination, and the left lung was excised. After dye extraction, fluorescence from lodged microspheres was determined (Fluorescence Spectrophotometer; Digilab, Holliston, MA) and normalized to total injected.

### Dexamethasone treatment

24 h prior to LPAL, rats were treated with the glucocorticoid dexamethasone-2-phospate (Sigma, D1159, 1 mg/kg iv) or its vehicle (saline, n = 4/group). This dose was selected based on the work of Hsieh [20] and adapted in preliminary experiments to the lowest effective dose required to limit ischemic injury (BAL protein). For evaluation of proliferating endothelium by histology, an additional dexamethasone treatment (1 mg/kg i.v.) was given 24 h after LPAL. For functional angiogenesis evaluated 14 d after LPAL, additional treatments were given 1, 4, (1 mg/kg i.v.), 7, 10, and 13 days (0.5 mg/kg i.v.) after LPAL.

### Statistical analysis

Results are presented as mean ± standard errors. Data were analyzed using the Kruskal-Wallis test, with post-hoc analysis by Dunn's multiple comparison test for all experiments except for blood flow measurement and % of changes after dexamethasone treatment (Mann-Whitney for unpaired comparisons), total protein with dexamethasone treatment (2-way ANOVA). Count data was transformed using the square root transform. P<0.05 was accepted as statistically significant.

## Results

### Obstruction of the pulmonary circulation induces changes in left lung parenchyma and left bronchus

**Figure 1:**
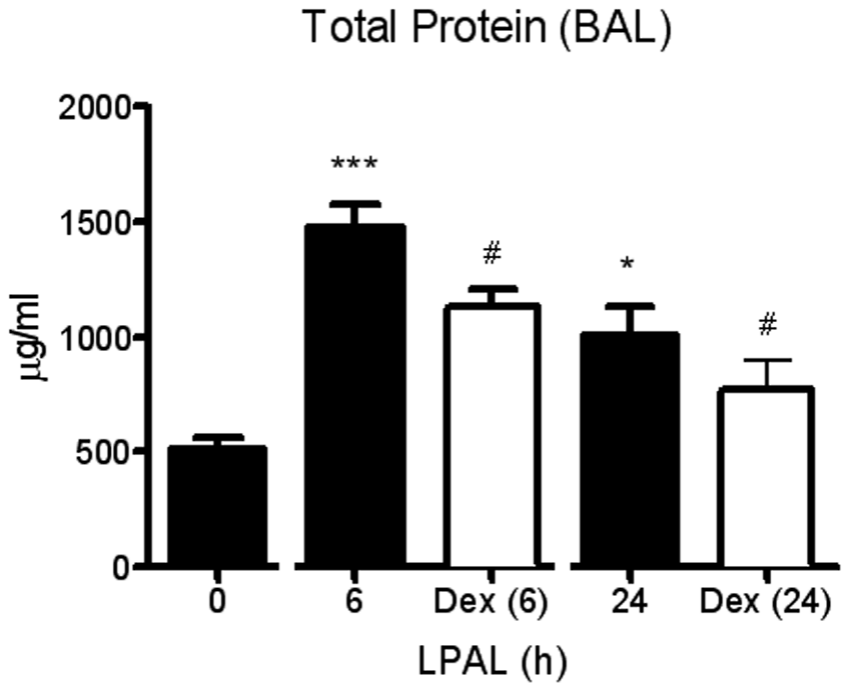
Time course of total protein in BAL during the initial 24 h following LPAL (BCA assay). An early increase occurred at 6 h LPAL (20 rats/group, ***P<0.001), and remained elevated at 24 h LPAL (18 rats/group, *P<0.05). Anti-inflammatory treatment with dexamethasone significantly decreased total protein content at 6 h and 24 h LPAL (11–12 rats/time point, ^#^P<0.05). *P measured vs 0 h LPAL, ^#^P measured vs time-matched untreated.

**Figure 2:**
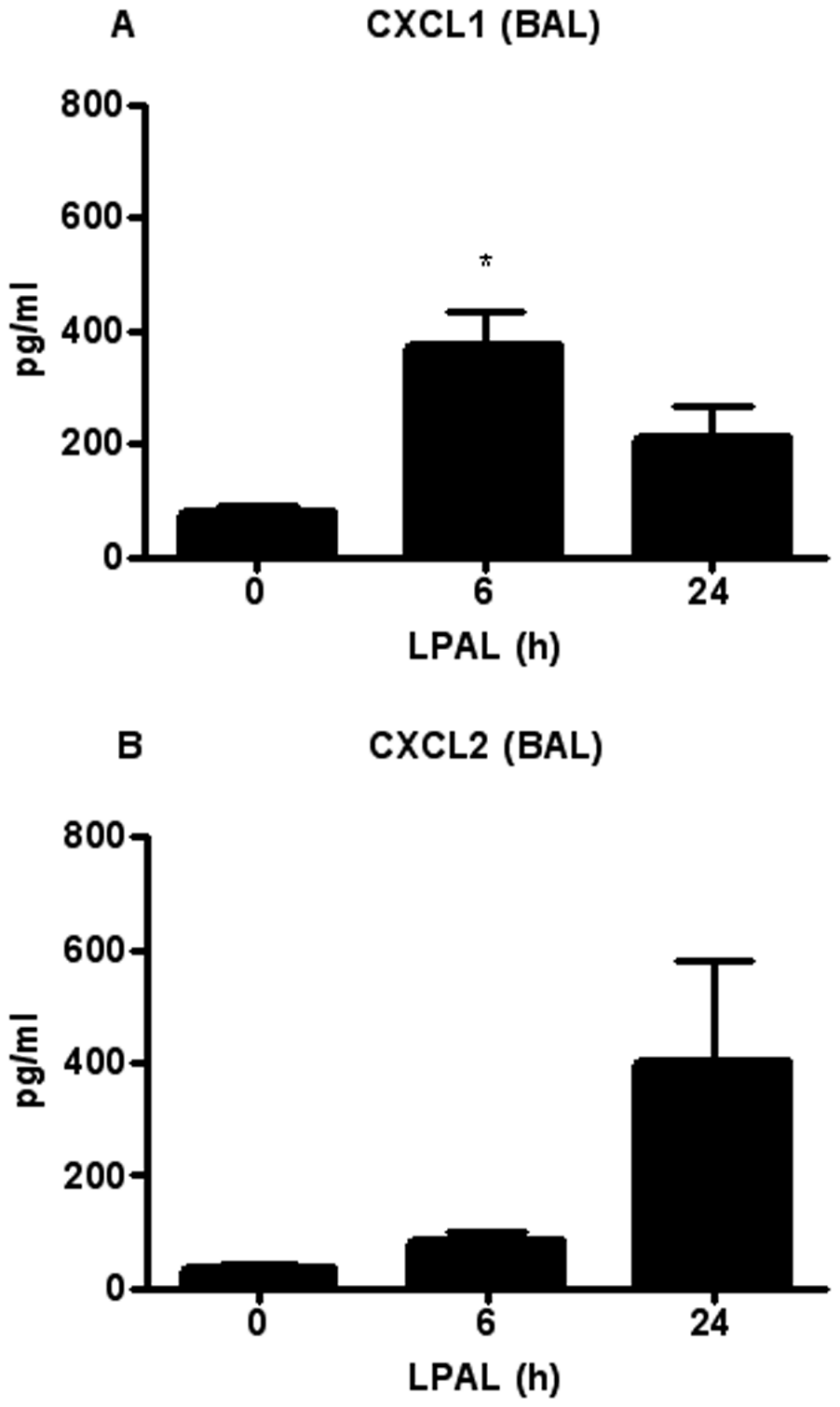
Time course of CXCL1 and CXCL2 cytokines in BAL. CXCL1 significantly increased at 6 h after LPAL, and decreased at 24 h LPAL (8–11 rats/time point, *P<0.05 vs 0 h). CXCL2 showed no significant changes (13 rats/time point).

**Figure 3:**
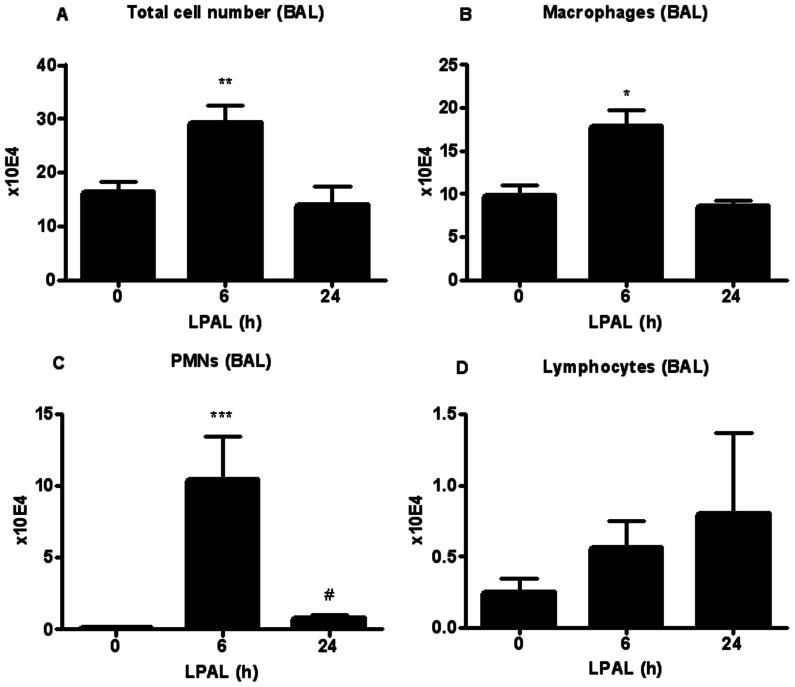
Inflammatory cell profile in BAL during the initial 24 h after LPAL. The total cell number (A) significantly increased at 6 h LPAL (10 rats/group, *P<0.05) then decreased at 24 h LPAL (3 rats/group), returning to baseline (0 h LPAL). The number of macrophages (B) (3–8 rats/group, *P<0.05), as well as the number of neutrophils/PMNs (6–11 rats/group, ***P<0.0001) followed the same trend. Lymphocytes (D) did not change during the same time course (3–7 rats/group). P measured vs 0 h LPAL.

As an indicator of lung injury and vascular permeability, the time course of changes in total protein (µg/ml) was measured in BAL immediately (0 h), 6 h and 24 h after LPAL (n = 6–19 rats/time point; P<0.0001 0 h vs 6 h, P<0.05 0 h vs 24 h). As shown in **Figure 1** the total protein content increased substantially by 6 h (300% increase) and remained significantly elevated at 24 h (200% increase), compared with 0 h control levels. To determine the acute inflammatory response within the initial 24 h after LPAL, the amount of the pro-inflammatory cytokines CXCL1 and CXCL2 (pg/ml) was also determined in BAL for the same time course. CXCL1 protein showed the same trend as the total protein content, reaching a maximum at 6 h LPAL (P<0.05) then decreasing towards baseline by 24 h LPAL. In contrast, CXCL2 trended toward increased levels by 24 h, however, these variable changes did not reach statistical significance (**Figure 2**). Total chemokine burden was roughly equivalent for CXCL1 and CXCL2 averaging approximately 400 pg/ml. The inflammatory cell profile in BAL for the same time course demonstrated a similar pattern with an early significant increase by 6 h and a return to the 0 h control level by 24 h (**Figure 3**). Evaluation of cell differentials demonstrated that the increase at 6 h was due to significant changes in the number of polymorphonuclear leukocytes (PMN; P<0.0005) representing an average 10000% and macrophages (P<0.05) representing an average of roughly 200%. Lymphocytes represented overall, a small percentage of total cells and did not significantly change during the first 24 h after LPAL.

**Figure 4:**
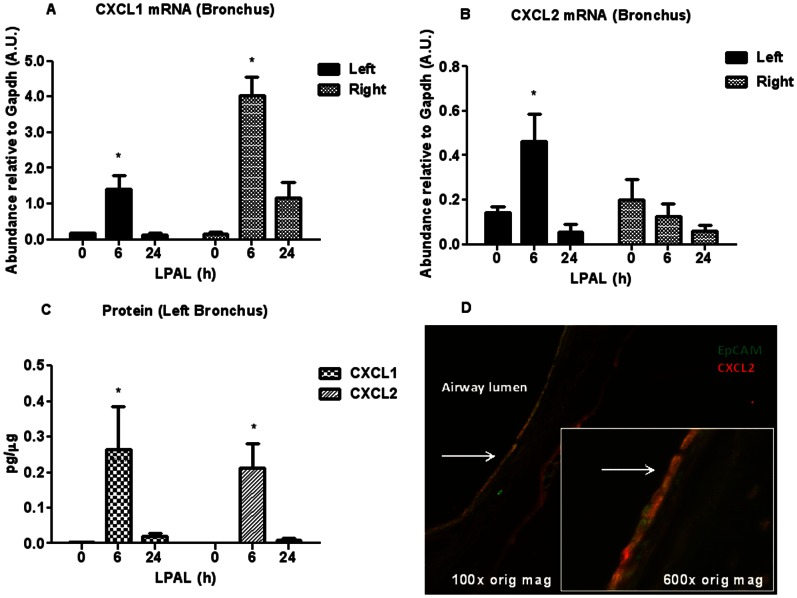
CXCL1 and CXCL2 cytokines mRNA (A, B), protein levels in the left bronchus (C), and (D) frozen section of left bronchus 6 h after LPAL with double staining for CXCL2 (red) and the epithelial cell marker Epcam (green;100× original magnification and inset: 600× original magnification). CXCL1 (A) and CXCL2 (B) mRNA and CXCL1 and CXCL2 protein levels (C) increased at 6 h LPAL (*P<0.05) and returned to baseline by 24 h LPAL (3–4 rats/time point). Co-localization of stain for epithelial cells (Epcam,;green) and anti-CXCL2 (red) suggest the airway epithelium is a prominent source for CXCL2.

To evaluate the bronchial tissue compartment directly, chemokine mRNA and protein were determined at the same time points (n = 3–4 rats/time point). To ensure specific responses due to left pulmonary artery ligation that might predict left lung angiogenesis, the time course of chemokine message was evaluated in both left and control right bronchi. CXCL1 gene expression increased significantly by 6 h in both the left and right bronchus (P<0.01) suggesting a non-specific response to surgery/anesthesia. However, only the left bronchus showed a significant increase in CXCL2 by 6 h after LPAL when compared to 0 h or to the right bronchus at 6 h (P<0.05). Both CXCL1 and CXCL2 protein were confirmed in left bronchial tissues by 6 h after LPAL (P<0.05). Pursuing the cell source of the specific CXCL2 protein in the left bronchus, frozen sections were obtained 6 h after LPAL. Figure 4D shows CXCL2 expression co-localized with Epcam, an airway epithelial cell marker.

**Figure 5:**
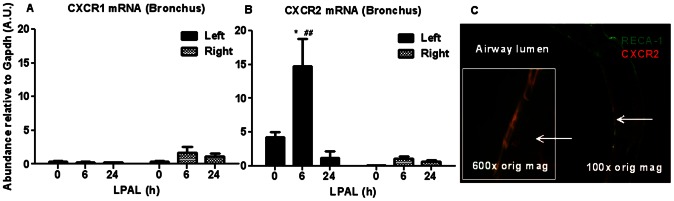
CXCR1(A) and CXCR2 (B) mRNA in left and right bronchi, and (C) co-localization of CXCR2 with RECA-1+ subepithelial blood vessel. Significant changes in CXCR2 were measured only in the left bronchus (*P<0.05 from 0 h and ##P<0.01 from right bronchus). Frozen sections of left bronchus 6 h after LPAL show co-localization of anti-CXCR2 (red) with RECA-1+ subepithelial blood vessels (green: 100× original magnification, and inset 600× original magnification).

Analysis of mRNA levels of the receptors CXCR1 and CXCR2, engaged when CXCL1 and CXCL2 signaling is activated, showed that only CXCR2 increased at 6 h in the left bronchus (300-fold increase, P<0.05). No changes occurred for CXCR1, or for either receptor in the right bronchus, suggesting the main role of CXCR2 in the early response after LPAL. Confirmation of CXCR2 co-localized with bronchial endothelium (RECA-1+) is provided in images obtained from frozen sections of the left bronchus 6 h after LPAL (**Figure 5C**).

### Treatment with dexamethasone limits pulmonary ischemic injury

**Figure 6:**
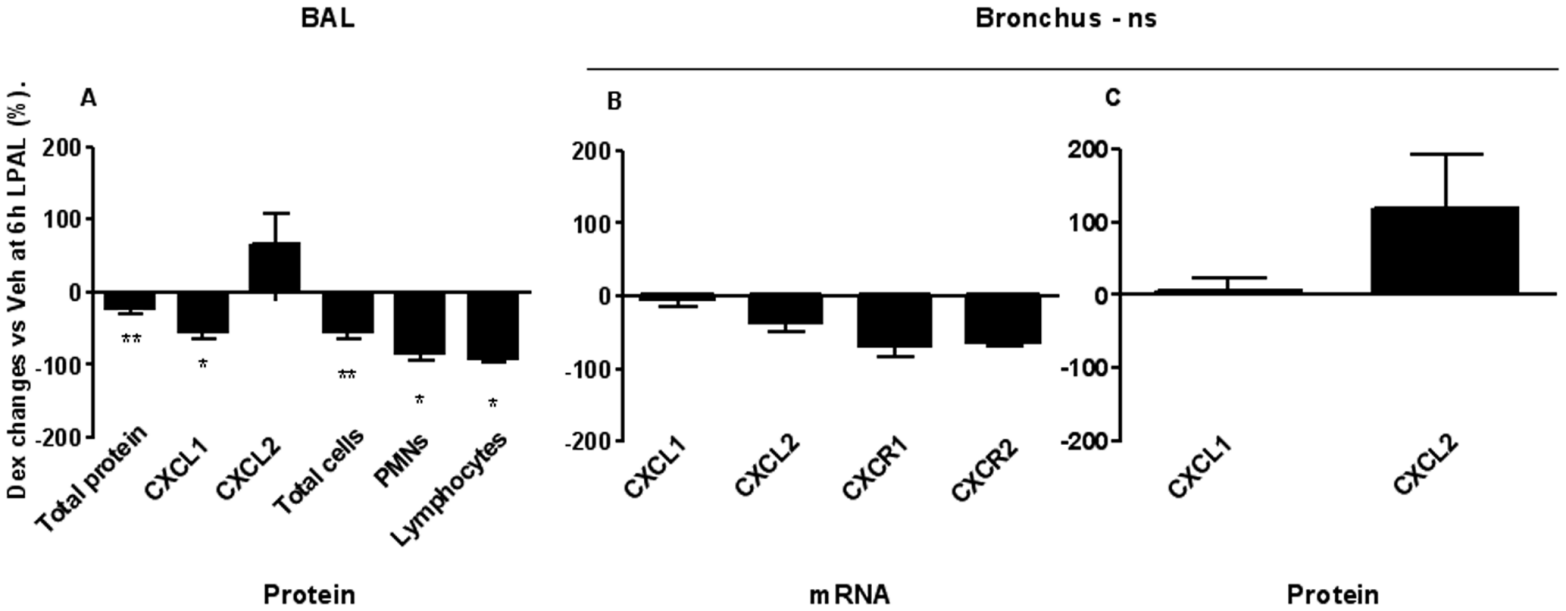
Effects of dexamethasone on parenchymal and airway cells and cytokines/receptors. (A) Summary of the average effect of dexamethasone on measured parameters reflecting changes within the alveolar space. Each bar represents the average % decrease 6 h after LPAL in dexamethasone treated rats compared with vehicle treated rats (3–11 rats/group, *P<0.05). B–C. Average effects of dexamethasone treatment on CXCL1, CXCL2, (mRNA, protein), CXCR1 and CXCR2 (mRNA) expression in the left bronchus 6 h after LPAL. Dexamethasone has no significant effects on any of the variables measured in the bronchus (3–4 rats/group).

Treatment with the anti-inflammatory dexamethasone reduced total protein extravasation in BAL as seen in **Figure 1** (11 rats/group). Both time (P<0.005) and treatment (P<0.016) had a significant effect on BAL protein between 6 h and 24 h, demonstrating a significant reduction in injury as assessed by permeability changes. When BAL CXCL1 and CXCL2 levels were measured in rats after dexamethasone treatment, CXCL1 protein was significantly reduced 6 h after LPAL (average 50% decrease; 7 rats/group, P<0.05). No additional changes were observed when compared to data presented in **Figure 2**. With regard to the inflammatory cell profile, dexamethasone treatment reduced total cell number in BAL at 6 h LPAL (50% reduction, 8 rats/group, P<0.05). This change was due to significant reductions in PMN (85% reduction; 9 rats/group, P<0.05) and lymphocytes (90% reduction; 9 rats/group, P<0.05). Significant effects of dexamethasone treatment on parameters evaluated in this study are summarized in **Figure 6A** and shown as percent changes from values 6 h after LPAL with vehicle treatment. However, dexamethasone treatment had no significant effect on CXCL1 or CXCL2 mRNA and protein in the left bronchus nor CXCR1 and CXCR2 mRNA expression in left bronchus (**Figure 6B and 6C**; 3–4 rats/group).

### Negative modulation of the inflammatory response is not sufficient to reduce bronchial angiogenesis

**Figure 7:**
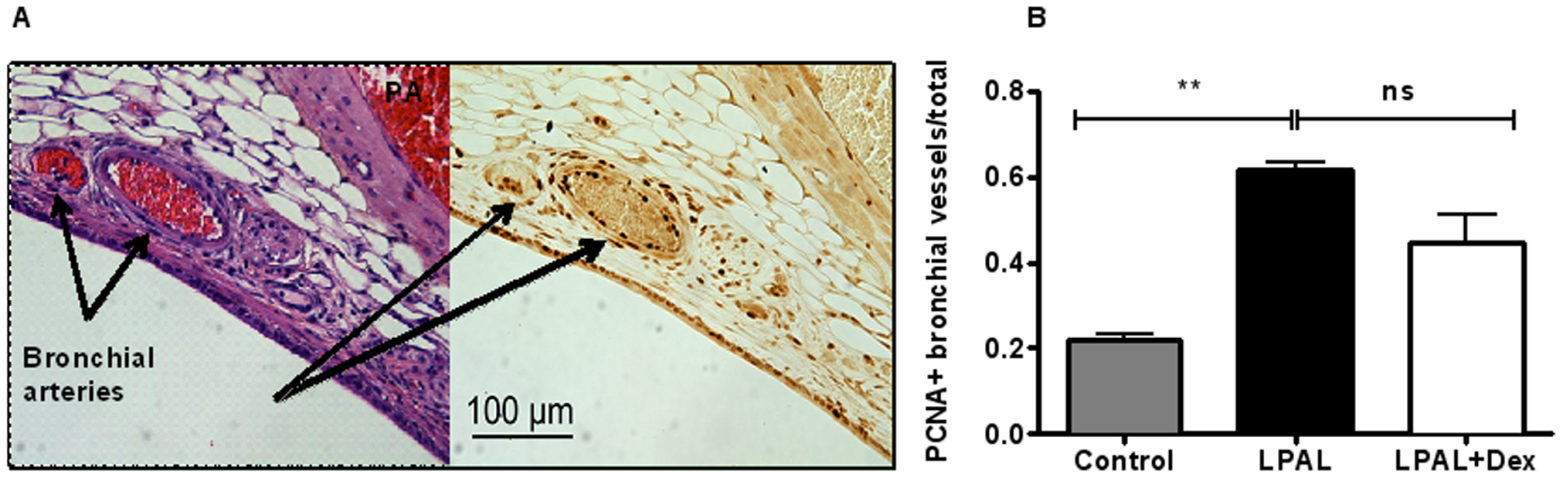
Changes in proliferating bronchial vessels. (A) Histologic section of airway demonstrating bronchial vessels by H&E (left panel) and serial section stained for PCNA (right panel). Bronchial vessels show abundant PCNA+ endothelial cells. (B) After LPAL (3 d), a significant increase in the fraction of PCNA^+^ bronchial vessels is observed. Treatment with dexamethasone had no significant effect on this proliferation index. Control includes both sham and right lungs (3–5 rats/group, **P<0.01 vs control).

**Figure 8:**
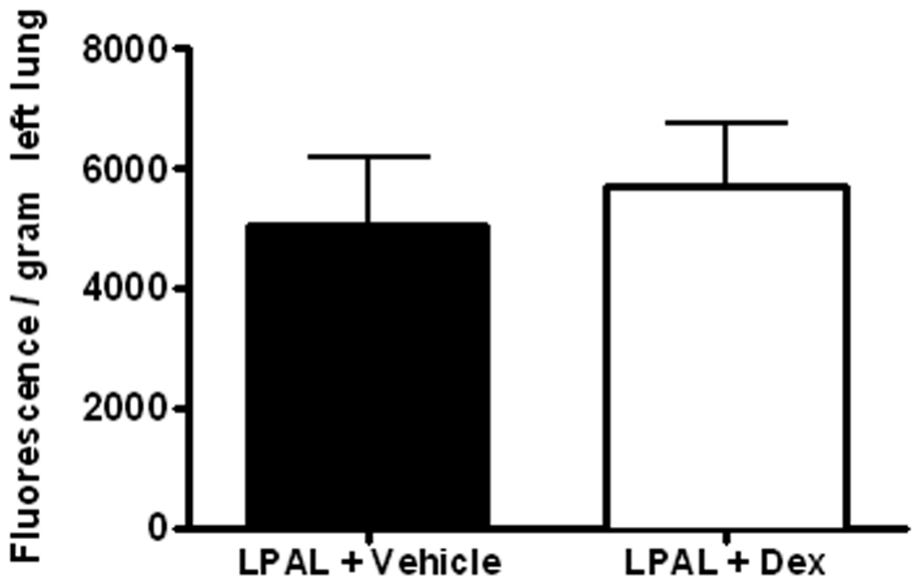
Functional angiogenesis assessed by fluorescent microsphere infusion 14 d after LPAL Dexamethasone treatment had no significant effect on the magnitude of bronchial angiogenesis (n = 4–5/group).

To determine if dexamethasone affected bronchial neovascularization after pulmonary ischemia, we measured proliferative status of bronchial vascular endothelium early (3 d, n = 3–5 lungs/group) and functional neovascularization later (14 d n = 4–5/group) after LPAL. **Figure 7A** shows a representative image (200× original magnification) of an airway wall showing bronchial vessels and associated positive PCNA staining. An average of 25±3 airways/lung were studied which were associated with an average of 205±20 bronchial vessels that were evaluated for bronchial endothelial cell proliferation. Control lungs represent left lungs from rats undergoing sham surgery (thoracotomy with no LPAL) and since they did not differ, were grouped with the right lungs of sham and LPAL rats. LPAL caused a significant increase in the fraction of PCNA^+^ vessels (P<0.008, **Figure 7B**). Treatment of rats with dexamethasone had no significant effect on the fraction of proliferating bronchial vessels assessed 3 d after LPAL (p>0.05 LPAL vs LPAL + dex). Functional blood vessels perfusing the left lung after LPAL were quantified by infusing fluorescent microspheres into the systemic circulation 14 d after LPAL. Fluorescence measured in the left lung after tissue harvest showed that dexamethasone treatment did not alter the functional perfusion (**Figure 8**). These rats had additional treatments throughout the 14 d time course. Despite this additional treatment, angiogenesis remained at the vehicle control level.

## Discussion

Angiogenesis in the lung is triggered by inflammatory conditions and specifically by up-regulation of the CXC chemokines [3], [22]–[25]. Their pro-angiogenic effect has been demonstrated in many studies [26]–[28] and work from our laboratory has shown that the cytokine CXCL2 and its high-affinity receptor CXCR2 are involved in the overall process of bronchial angiogenesis following pulmonary ischemia. This past study demonstrated an increase in total lung CXCL2 within the first day after the onset of ischemia and an inhibitory effect of anti-CXCR2 on bronchial angiogenesis [12], [29]. Yet the localization within the lung and the temporal regulation of the CXC chemokines that lead to bronchial arteriogenesis are unknown. Moreover, it is unclear how lung parenchymal growth factors can activate upstream bronchial angiogenesis. Thus, the present study was designed to examine the early expression of CXC chemokines following pulmonary ischemia (0–24 h), specifically focusing on the timing and local expression of CXCL1 and CXCL2 in BAL fluid and airway tissue. Additionally, we studied the effectiveness of anti-inflammatory therapy in limiting local cytokine levels (BAL and airway tissue), and to correlate this effect with the magnitude of bronchial angiogenesis. Our data demonstrate the presence of both chemokines within both lung compartments. However, the CXCL1 and CXCL2 modulation pattern was different within the two locations analyzed. CXCL1 release appears to be more generally associated with injury and anesthesia/surgery. Furthermore, anti-inflammatory treatment affected only the extent of lung injury, BAL CXCL1 level and BAL inflammatory cells. No changes were observed in the level of chemokine expression within bronchial tissue or, importantly, the magnitude of angiogenesis. In conclusion, results suggest that early changes within the bronchial niche where arteriogenesis originates, contribute to subsequent neovascularization during pulmonary ischemia.

Our initial experiments evaluated BAL content as an index of predominantly parenchymal responses. In this model, we hypothesized that alveolar fluid could redistribute and act as a conduit for growth factors, through mucociliary movement. We demonstrated that acute ischemia caused an early increase in BAL total protein suggesting acute lung injury. These permeability changes were substantial (3-fold increase), early, and seemed to be attenuated by 24 h after the onset of ischemia. We had shown previously that permeability changes were again seen 14 d after LPAL [12], [29]. In addition, the inflammatory cell response paralleled the protein leak and likely reflected resident cells because of the lack of pulmonary blood flow, and only the small bronchial vasculature at this time point. Specifically, the inflammatory cell profile showed a predominance of neutrophils and macrophages, which, in addition to epithelial cells, have the capacity to secrete both CXCL1 and CXCL2 chemokines [30]. Although CXCL1 showed a reproducible increase by 6 h after LPAL, changes in BAL CXCL2 were inconsistent. Measurements of CXCL1 were included since recent studies suggest that both chemokines can play a role in angiogenesis signaling [31], [32]. However, given the pattern of protein and inflammatory cell changes at the three time points (0, 6 and 24 h), the appearance of CXCL1 seems to reflect injury. That we did not see robust early increases in CXCL2 was somewhat surprising since in our previous study, left lung homogenate showed a substantial increase between 4 and 24 h [12]. This observation suggests that BAL fluid may not fully reflect lung parenchymal changes.

In an attempt to examine more specifically the niche where bronchial vessels originate, we also studied the tissue comprising the left mainstem bronchus. We evaluated CXCL1 and CXCL2 cytokine messenger RNA and protein expression within the bronchial tissue. Results showed that both CXCL1 and CXCL2 protein expression was increased by 6 h after the onset of ischemia. However, since we saw that the right bronchus also showed increased CXCL1 gene expression, we suggest that this is a more generalized response to surgery and or anesthesia. Interestingly, CXCL2 and its primary receptor CXCR2 were uniquely expressed only in the left bronchus, the site of subsequent arteriogenesis. This observation suggests that CXCL2 secretion by local cells within the left bronchus could serve a paracrine role and initiate the growth cascade. Immunostaining for CXCL2 demonstrated that epithelial cells are a likely source of chemokine contributing to the measured changes. However, the contribution of local inflammatory cells, specifically macrophages and neutrophils, requires further evaluation. How pulmonary ischemia stimulates the up-regulation of CXCL2 within large airways is still mechanistically unknown. Given the increase observed in CXCL2 and its epithelial cell source, it is unclear why an increase in this chemokine was not seen in the BAL fluid. This apparent inconsistency might suggest the existence of a positive feedback among members of the CXC family. For example, Zhang and colleagues showed different compartmentalization of CXCL1 and CXCL2 in a rat model of intratracheal LPS challenge [33]. Their results implied that individual CXC chemokines are specifically regulated and may have different effects in the alveolar space and bronchial tissue. Moreover, Zamjahn and coworkers have shown that CXCL1 clearance rate from plasma and blood was more than sevenfold and fourfold higher, respectively, than CXCL2. This indicates the presence of a different flux of CXCL1 and CXCL2 across the epithelial/endothelial barrier of the lung, despite similar molecular size [34]. Cai and colleagues showed that CXCL1 can be important for the local expression of CXCL2 and other cytokines such as CXCL5 [35]. These data add other members of the CXC family to a complex scenario. Additional complexity is added when considering receptor affinities as CXCL2 was shown to have a 72-fold greater affinity for CXCR2 than CXCL1 [36]–[38]. Our results demonstrate unique up-regulation of CXCL2 and its predominant receptor CXCR2 in the left bronchus adding further support that this cytokine-receptor pair are important in airway remodeling.

Finally, we delivered an anti-inflammatory treatment to reduce the initial inflammatory response, in an attempt to link an attenuation in chemokine expression with the angiogenic response. Systemic inflammation has been shown to be effectively reduced by the glucocorticoid dexamethasone. Specifically, Sevaljevic and coworkers have demonstrated that dexamethasone treatment negatively modulates pro-inflammatory CXC chemokines [39]. Moreover, several authors have proved its anti-angiogenic effect [40]–[42]. In particular, Nakao and collaborators have shown that dexamethasone (10 mg/kg i.p.) inhibits vascular growth in a model of inflammatory corneal angiogenesis [41]. Also, Yao and colleagues have demonstrated that it reverses tracheal vessel remodeling in Mycoplasma pulmonis-infected mice (4.8 mg/day, eye drop) [42]. In the LPAL model, pretreatment with dexamethasone was effective in reducing ischemic injury at a dose even lower (1 mg/Kg) than what was previously shown to be effective in down-regulating chemokine levels [20]. However, only BAL chemokines and inflammatory cells were affected. No changes in the level of chemokine expression within bronchial tissue or, importantly, the magnitude of angiogenesis, were observed. Specifically, dexamethasone treatment resulted in significantly reduced BAL total protein, total inflammatory cells, CXCL1 protein, but not CXCL2 or macrophages. Chemokine expression in the bronchus was unchanged. Furthermore, no effects were observed at later time points either in bronchial endothelial proliferation (3 d) or bronchial perfusion of the lung (14 d). Previously, we have measured angiogenesis at late time points when a functional, perfusing vasculature was established. Although we applied the fluorescent microsphere technique in the current study as well, we also examined bronchial endothelial cell proliferation 3 d after LPAL. This additional approach aimed to quantify early angiogenesis through airway morphometry. Results showed bronchial vessels after LPAL with consistently higher numbers of PCNA^+^ endothelial cells than sham controls or right bronchi. However, dexamethasone treatment did not reduce such endothelial cell proliferation, nor altered angiogenesis, even with the previously shown effectiveness in limiting BAL fluid components that had access to bronchial vessels. Furthermore, early signals occurring within the bronchial niche where arteriogenesis originates, appeared not to be altered either. Based on these results, we suggest that the local bronchial tissue environment plays a critical role in inducing specific growth factors important for subsequent neovascularization during pulmonary ischemia.

In summary, our results confirm the presence of CXC chemokines within BAL fluid as well as within the left mainstem bronchus. Despite significant reduction in lung injury and inflammation with dexamethasone treatment, both CXCL1 and CXCL2 chemokine expression within the bronchial tissue as well as angiogenesis were not affected. We conclude that early and specific changes within the bronchial niche selectively contribute to subsequent neovascularization during pulmonary ischemia.
